# Varicella-Zoster Virus Infections in Pediatric Malignancy Patients: A Seven-Year Analysis

**DOI:** 10.4274/tjh.2016.0046

**Published:** 2016-12-01

**Authors:** Mine Düzgöl, Gülcihan Özek, Nuri Bayram, Yeşim Oymak, Ahu Kara, Bengü Demirağ, Tuba Hilkay Karapınar, Yılmaz Ay, Canan Vergin, İlker Devrim

**Affiliations:** 1 Dr. Behçet Uz Children Training and Research Hospital, Clinic of Pediatric Infectious Diseases, İzmir, Turkey; 2 Dr. Behçet Uz Children Training and Research Hospital, Clinic of Pediatric Hematology and Oncology, İzmir, Turkey

**Keywords:** Varicella, malignancy, Pediatric patient

## Abstract

Primary varicella-zoster virus (VZV) infection is a benign self-limited disease. In this study, we review our experience in focusing on the outcome and treatment of VZV infection in pediatric malignancy patients. During the study period, a total of 41 patients with pediatric malignancy had been hospitalized with the diagnosis of VZV infection. All the patients were treated with intravenous acyclovir for a median of 7 days (ranging from 5 to 21 days). The calculated attributable delay of chemotherapy due to VZV infections was 8 days (ranging from 2 to 60 days). VZV-related complications were observed in 3 of 41 patients (7%) who suffered from acute respiratory distress syndrome, and one of them with hemophagocytic lymphohistiocytosis died due to respiratory failure despite acyclovir and broad-spectrum antimicrobial treatment plus supportive treatment. VZV infections are still important contagious diseases in pediatric cancer patients, because they cause not only significant mortality but also a delay in chemotherapy.

## Abstract (tr)

Primer varisella zoster virüs (VZV) enfeksiyonu benign, kendi kendini sınırlayan bir hastalıktır. Bu çalışmada pediatrik malignitesi olan hastalarda VZV enfeksiyonu ve tedavisine odaklı tecrübelerimizi gözden geçirmeyi amaçladık. Çalışma süresi boyunca; VZV enfeksiyonu tanısı alan pediatrik maligniteli toplam 41 hasta hastaneye yatırıldı. Tüm hastalar ortalama 7 gün (5 ila 21 gün arasında değişen) intravenöz asiklovir ile tedavi edildi. VZV enfeksiyonlarına bağlı olarak hesaplanan atfedilebilir kemoterapi gecikmesi ortalama 8 gündü (2 ile 60 gün arasında değişen). VZV enfeksiyonuna bağlı komplikasyonlar 41 hastadan 3’ünde (%7) akut solunum distres sendromu olarak görüldü ve bu hastalardan hemofagositik lenfohistiyositozu olan bir tanesi asiklovir, geniş spektrumlu antibiyotik ve destekleyici tedaviye rağmen solunum yetmezliği nedeniyle kaybedildi. VZV enfeksiyonları, pediatrik malignite hastalarında hala önemli bulaşıcı hastalıklardan biridir, çünkü sadece ciddi mortaliteye sebep olmakla kalmayıp kemoterapi başlangıcını da geciktirmektedir.

## INTRODUCTION

Immunocompromised children are at greater risk of suffering from severe, prolonged, and complicated varicella-zoster virus (VZV) infection [1]. Before introduction of antiviral therapy, the mortality rate of VZV infections in children with cancer was reported to be 7%, with numbers reaching up to 55% in cases with visceral involvement [2,3,4,5]. In this study, we aimed to review our experience in focusing on the outcome and treatment of VZV infections in pediatric malignancy patients.

## MATERIALS AND METHODS

A retrospective cohort study design was used to evaluate pediatric cancer patients with VZV infections who were hospitalized in the Pediatric Hematology-Oncology and Infectious Diseases Units of the Dr. Behçet Uz Children’s Hospital from December 2008 to March 2015. In this study, the attending physician’s clinical diagnosis of VZV infection was based on case definitions set by the United States Centers for Disease Control and Prevention and the Council of State and Territorial Epidemiologists guidelines reported in 2009 [6,7]. Therapy with intravenous acyclovir (1500 mg/m2/day) in 3 divided doses was started on the first day of the onset of rash. VZV infection-related complications were defined as a condition or event occurring within 14 days of the onset of VZV infection [2]. Statistical analysis was done using SPSS 16.0 (SPSS Inc., Chicago, IL, USA).

## RESULTS

During the study period, a total of 41 patients with pediatric malignancy had been hospitalized with the diagnosis of VZV infection. Among them, 14 (34.1%) were female and 27 (65.9%) were male. The mean age was 58.8±32.4 months (within the range of 8 months to 12 years of age). Of the patients, 29 had acute lymphoblastic leukemia (ALL) (70.7%), followed by 2 cases of acute myeloblastic leukemia (4.9%), 3 cases of Wilms tumor (7.3%), 2 cases of hemophagocytic lymphohistiocytosis (HLH) (4.9%), 2 cases of rhabdomyosarcoma (4.9%), 2 cases of neuroblastoma (4.9%), and 1 case of hepatoblastoma (2.4%). Among the ALL patients, 8 (27.5%) of them were in the induction phase of chemotherapy (ALL REZ-Berlin-Frankfurt-Münster protocol), 19 (65.5%) of them were in a maintenance phase, and 2 patients (6.8%) had relapsed ALL. Only 2 children (4.9%) had a known exposure to siblings in the household who had developed chickenpox.

Among the 41 patients, neutropenia was present in 18 patients (43.9%), lymphopenia was present in 27 (65.9%) patients, thrombocytopenia was present in 10 patients (24.4%), and anemia was present in 23 (56.1%) patients. Twenty-one patients had associated fever at the time of diagnosis of VZV infection. Active vesicular rashes were present in all of the patients at the time of diagnosis and the median duration of active VZV infection was 7 days (ranging from 5 to 21 days). All patients had been admitted to our hospital within the first day of the onset of rash.

All the patients were treated with intravenous acyclovir for a median of 7 days (ranging from 5 to 21 days). During acyclovir treatment, no serious adverse effects including elevation in blood creatinine and urea levels or hematuria were observed, while 2 patients (4.8%) had nausea and vomiting that could not be explained with other reasons.

The median hospital stay was 7 days (ranging from 3 to 35 days) and the calculated attributable delay of chemotherapy due to VZV infections was 8 days (ranging from 2 to 60 days). Thirty-eight patients (93%) showed no complications, but 3 patients (7%) suffered from Acute respiratory distress syndrome (ARDS). Two of them required mechanical ventilation and one required noninvasive ventilation; the patient with HLH (1%) died due to respiratory failure despite acyclovir and broad-spectrum antimicrobial treatment plus supportive treatment.

## DISCUSSION

Secondary attack rates among susceptible household contacts of people with VZV are as high as 90%; i.e. 9 out of 10 susceptible household contacts will become infected [8]. In this study, only 2 children (4.9%) had a known exposure to siblings in the household who had developed chickenpox. The majority of the patients had no known exposure; it was reported that for half of the ALL cases with varicella infections, the source of infection was unknown [9]. Our findings suggest that, regarding the high secondary attack rates of VZV infection, precautions should be taken for preventing possible contact of malignancy patients with VZV patients, especially in outpatient clinics including elevators, playgrounds, etc.

In our study, the most common underlying malignant disease was ALL (70.7%), supporting the findings of a previous report [10]. Patients with an underlying diagnosis of ALL and children less than 5 years of age were reported to develop complications more than any other age group, which was consistent with other studies [2]. In our study the ages of the most complicated cases were above 5 years, which showed that patients in every age group were at risk of serious VZV infection.

Immunocompromised patients develop serious complications, such as secondary bacterial infection with invasive Streptococcus pyogenes [11]. However, in our study, we experienced Streptococcus pneumoniae sepsis only in one ALL patient who required noninvasive mechanical ventilation support. In our study, our patients who underwent intensive chemotherapy faced complications and even death. Previous reports showed higher mortality rates than our study, such as 7% in 60 patients who were undergoing chemotherapy due to primary VZV pneumonitis, with or without acute encephalitis [11]. Before the introduction of specific antiviral therapy, the mortality rate of VZV infections in children with cancer was reported to be 7%-10%, with rates reaching up to 55% in cases with visceral involvement [2,3,4,5]. Children with acute leukemia who had VZV infections were reported to have a high risk for VZV pneumonia, which might occur in up to one-third of patients with a fatality rate of about 10% [12]. In our study, three patients (7%) with low absolute neutrophil count suffered from ARDS and one of them died because of respiratory failure. The fatality rate was about 2%.

Our study showed that the complicated cases were not homogeneously distributed regarding their primary diseases. This visceral dissemination was thought not be related to the type or status of the malignancy or to the duration of specific anticancer therapy. VZV was more likely to disseminate in children with absolute lymphopenia, less than 500 cells per cubic millimeter, than in patients with higher lymphocyte counts. Patients with lymphopenia or poor cell-mediated immune responses during VZV infection are said to be at risk for persistent, severe, or even fatal VZV [13]. Our patients with complicated clinical features had lymphopenia and neutropenia, suggesting a correlation between immune status and poor outcome.

Immunocompromised children, particularly those with leukemia, have more numerous lesions, often with a hemorrhagic base, and healing takes nearly three times longer than in healthy children with VZV infections. These patients were reported to suffer from severe progressive VZV infections characterized by continuing eruption of lesions and high fever persisting into the second week of illness [14]. In our study, despite the median duration of the active chickenpox rash being 7 days, in some cases active hemorrhagic vesicular lesions were observed until 21 days of disease. During our study the median hospital stay was 7 days, similar to a previous report of 7.96±3.57 days [13]. Effective treatment with acyclovir is thought to be a significant factor in reducing the severity and mortality of infection [15]; however, mortality is not the only problem with cancer patients. One of the most important findings in our study was that, regardless of the primary disease and chemotherapy protocol, chemotherapy was delayed for at least for 2 days with a median of 8 days, which could cause undesirable effects on the overall chemotherapy protocol in children.

In conclusion, VZV infections are still important contagious diseases in pediatric cancer patients because they cause not only significant mortality but also a delay in chemotherapy. Thus, infection control preventions should be taken in hospitals and maximum efforts for preventing possible exposure of pediatric cancer patients to VZV-infected children should be made.

## Ethics

Ethics Committee Approval: Retrospective study; Informed Consent: Retrospective study.
